# 2,2′-Bis(meth­oxy­meth­oxy)-3,3′-diphenyl-1,1′-binaphthalene

**DOI:** 10.1107/S1600536810053018

**Published:** 2010-12-24

**Authors:** Hua Zong, Hua-Yin Huang, Bing Hu, Guang-Ling Bian, Ling Song

**Affiliations:** a State Key Laboratory of Structural Chemistry, Fujian Institute of Research on the Structure of Matter, Chinese Academy of Sciences, Fuzhou, Fujian 350002, People’s Republic of China

## Abstract

The asymmetric unit of the title compound, C_36_H_30_O_4_, contains two crystallographically independent mol­ecules of similar geometry. In both mol­ecules, the meth­oxy­meth­oxy groups are disordered over two positions with refined site occupancies of 0.613 (3):0.387 (3) and 0.589 (4):0.411 (4). The dihedral angles between the naphthalene planes within the same mol­ecule are 71.72 (7) and 71.73 (8)°. In the crystal, neighbouring mol­ecules are linked by inter­molecular C—H⋯O hydrogen bonds, forming double chains parallel to the *c* axis.

## Related literature

For the application of 1,1′-bi-2-naphthol derivatives in asymmetric syntheses, see: Lou *et al.* (2006 ▶); Brunel (2006 ▶). For the synthesis of the title compound, see: Wu *et al.* (2004 ▶). For bond-length data, see: Allen *et al.* (1987 ▶).
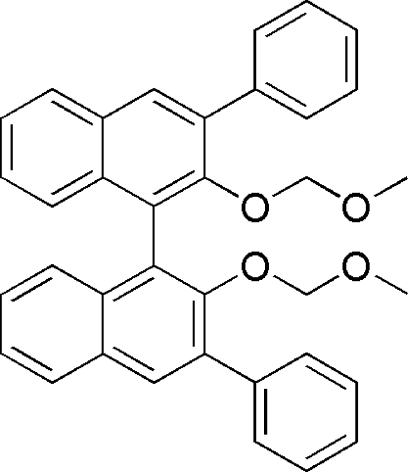


## Experimental

### 

#### Crystal data

C_36_H_30_O_4_
*M* *_r_* = 526.60
Monoclinic, 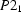

*a* = 11.3166 (3) Å
*b* = 19.4841 (3) Å
*c* = 14.0155 (4) Å
β = 110.172 (3)°
*V* = 2900.77 (12) Å^3^
*Z* = 4
Mo *K*α radiation
μ = 0.08 mm^−1^
*T* = 295 K
0.43 × 0.40 × 0.36 mm

#### Data collection

Oxford Xcalibur Eos CCD diffractometer
Absorption correction: multi-scan (*CrysAlis CCD*; Oxford Diffraction, 2006 ▶) *T*_min_ = 0.828, *T*_max_ = 1.000
12553 measured reflections
6211 independent reflections
4315 reflections with *I* > 2σ(*I*)
*R*_int_ = 0.020

#### Refinement

*R*[*F* ^2^ > 2σ(*F* ^2^)] = 0.036
*wR*(*F* ^2^) = 0.069
*S* = 1.01
6211 reflections
769 parameters
1 restraint
H-atom parameters constrained
Δρ_max_ = 0.16 e Å^−3^
Δρ_min_ = −0.20 e Å^−3^

### 

Data collection: *CrysAlis CCD* (Oxford Diffraction, 2006 ▶); cell refinement: *CrysAlis CCD*; data reduction: *CrysAlis RED* (Oxford Diffraction, 2006 ▶); program(s) used to solve structure: *SHELXS97* (Sheldrick, 2008 ▶); program(s) used to refine structure: *SHELXL97* (Sheldrick, 2008 ▶); molecular graphics: *DIAMOND* (Brandenburg & Berndt, 1999 ▶); software used to prepare material for publication: *SHELXL97*.

## Supplementary Material

Crystal structure: contains datablocks I, global. DOI: 10.1107/S1600536810053018/rz2537sup1.cif

Structure factors: contains datablocks I. DOI: 10.1107/S1600536810053018/rz2537Isup2.hkl

Additional supplementary materials:  crystallographic information; 3D view; checkCIF report

## Figures and Tables

**Table 1:** Hydrogen-bond geometry (Å, °)

*D*—H⋯*A*	*D*—H	H⋯*A*	*D*⋯*A*	*D*—H⋯*A*
C3—H3*A*⋯O6*A*^i^	0.93	2.59	3.448 (6)	153
C43—H43*A*⋯O2*B*^ii^	0.93	2.57	3.304 (7)	136
C51—H51*A*⋯O1	0.93	2.38	3.233 (3)	152

Symmetry codes: (i) 
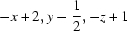
; (ii) 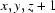.

**Fig. 1.**
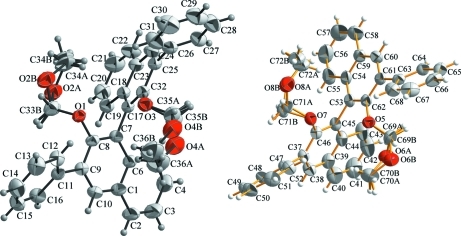
The molecular structure of independent molecules of the title compound, with atom labels and 50% probability displacement ellipsoids for non-H atoms.

**Fig. 2.**
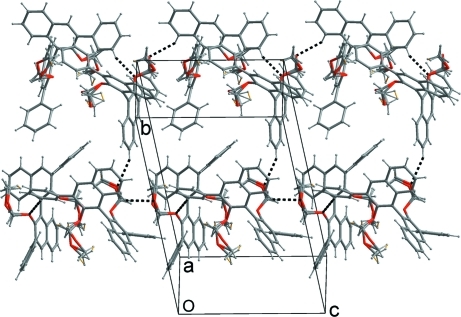
Crystal packing of the title compound showing the formation of double chains through intermolecular hydrogen bonds (dashed lines).

## supplementary crystallographic information

### Comment

Chiral compounds especially when used as chiral ligands are particularly important in asymmteric synthesis. 1,1'-Bi-2-naphthol (BINOL) and its derivatives have been widely used in asymmetric synthesis (Lou *et al.*, 2006; Brunel, 2006), and used as effective chiral ligands for various metal complex catalysis. As part of our research in this field, we synthesized the title compound, whose X-ray crystal structure is reported herein.

The asymmetric unit of the title compound contains two crystallographically independent molecules (Fig. 1). In both molecules, the methoxymethoxy groups are disordered over two positions with refined site occupancies of 0.613 (3):0.387 (3) and 0.589 (4):0.411 (4). Bond lengths (Allen *et al.*, 1987) and angles are within normal ranges. The dihedral angles between the naphthalene planes belonging to the same molecule are 71.72 (7)° and 71.73 (8)°. In the crystal structure, intramolecular C—H···O hydrogen bonds (Table 1) assemble neighbouring molecule into double chains running parallel to the *c* axis (Fig. 2).

### Experimental

The title compound was prepared by the reaction of (*R*,*R*)-3,3'-dibromo-2,2'-bis(methoxymethoxy)-1,1'-binaphthalene and phenylboronic acid according to the literature method (Wu *et al.*, 2004). Crystals suitable for X-ray analysis were obtained by dissolving the title compound (1.5 g) in ethyl acetate (20 ml) and evaporating the solvent slowly at room temperature for about 28 h.

### Refinement

All methoxymethoxy groups of the two molecules were refined as disordered, with site occupancies of 0.613 (3):0.387 (3) for the major and minor components respectively of O2/C33/C34, and of 0.589 (4):0.411 (4) for the major and minor components respectively of O4/C35/C36. During the refinement, the displacement parameters of the two components of the disordered atoms were set equal to each other. The H atoms were placed at calculated positions (C—H = 0.93 Å for aromatic, 0.97 Å for methylene and 0.96 Å for methyl H atoms), and were included in the refinement in the riding model approximation, with *U*_iso_(H) = 1.2*U*_eq_(C) or 1.5*U*_eq_(C) for methyl H atoms. In the absence of significant anomalous scattering effects, 4186 Friedel pairs were merged.

### Crystal data

**Table:**

C_36_H_30_O_4_	*F*(000) = 1112
*M**_r_* = 526.60	*D*_x_ = 1.206 Mg m^−^^3^
Monoclinic, *P*2_1_	Mo *K*α radiation, λ = 0.71073 Å
Hall symbol: P 2yb	Cell parameters from 6318 reflections
*a* = 11.3166 (3) Å	θ = 2.6–28.8°
*b* = 19.4841 (3) Å	µ = 0.08 mm^−^^1^
*c* = 14.0155 (4) Å	*T* = 295 K
β = 110.172 (3)°	Block, colourless
*V* = 2900.77 (12) Å^3^	0.43 × 0.40 × 0.36 mm
*Z* = 4	

### Data collection

**Table:**

Oxford Xcalibur Eos CCD diffractometer	6211 independent reflections
Radiation source: fine-focus sealed tube	4315 reflections with *I* > 2σ(*I*)
graphite	*R*_int_ = 0.020
Detector resolution: 16.2083 pixels mm^-1^	θ_max_ = 26.6°, θ_min_ = 2.6°
ω scans	*h* = −14→12
Absorption correction: multi-scan (*CrysAlis CCD*; Oxford Diffraction, 2006)	*k* = −24→24
*T*_min_ = 0.828, *T*_max_ = 1.000	*l* = −10→17
12553 measured reflections	

### Refinement

**Table:**

Refinement on *F*^2^	Primary atom site location: structure-invariant direct methods
Least-squares matrix: full	Secondary atom site location: difference Fourier map
*R*[*F*^2^ > 2σ(*F*^2^)] = 0.036	Hydrogen site location: inferred from neighbouring sites
*wR*(*F*^2^) = 0.069	H-atom parameters constrained
*S* = 1.01	*w* = 1/[σ^2^(*F*_o_^2^) + (0.015*P*)^2^ + 0.5*P*] where *P* = (*F*_o_^2^ + 2*F*_c_^2^)/3
6211 reflections	(Δ/σ)_max_ = 0.009
769 parameters	Δρ_max_ = 0.16 e Å^−^^3^
1 restraint	Δρ_min_ = −0.20 e Å^−^^3^

### Special details

**Table:**

Geometry. All e.s.d.'s (except the e.s.d. in the dihedral angle between two l.s. planes) are estimated using the full covariance matrix. The cell e.s.d.'s are taken into account individually in the estimation of e.s.d.'s in distances, angles and torsion angles; correlations between e.s.d.'s in cell parameters are only used when they are defined by crystal symmetry. An approximate (isotropic) treatment of cell e.s.d.'s is used for estimating e.s.d.'s involving l.s. planes.
Refinement. Refinement of *F*^2^ against ALL reflections. The weighted *R*-factor *wR* and goodness of fit *S* are based on *F*^2^, conventional *R*-factors *R* are based on *F*, with *F* set to zero for negative *F*^2^. The threshold expression of *F*^2^ > σ(*F*^2^) is used only for calculating *R*-factors(gt) *etc*. and is not relevant to the choice of reflections for refinement. *R*-factors based on *F*^2^ are statistically about twice as large as those based on *F*, and *R*– factors based on ALL data will be even larger.

### Fractional atomic coordinates and isotropic or equivalent isotropic displacement parameters (Å^2^)

**Table:**

	*x*	*y*	*z*	*U*_iso_*/*U*_eq_	Occ. (<1)
O1	0.66287 (16)	0.31954 (8)	0.06996 (12)	0.0407 (4)	
O3	0.85866 (16)	0.34414 (9)	0.28820 (13)	0.0488 (5)	
C1	0.8423 (3)	0.12956 (14)	0.1334 (2)	0.0456 (7)	
C2	0.9041 (3)	0.06530 (15)	0.1496 (2)	0.0613 (8)	
H2A	0.8569	0.0252	0.1391	0.074*	
C3	1.0311 (3)	0.06144 (17)	0.1803 (3)	0.0746 (10)	
H3A	1.0706	0.0188	0.1898	0.089*	
C4	1.1029 (3)	0.12118 (18)	0.1977 (3)	0.0725 (10)	
H4A	1.1901	0.1181	0.2180	0.087*	
C5	1.0474 (3)	0.18406 (15)	0.1854 (2)	0.0566 (8)	
H5A	1.0970	0.2234	0.2002	0.068*	
C6	0.9143 (2)	0.19010 (13)	0.1501 (2)	0.0419 (6)	
C7	0.8511 (2)	0.25519 (12)	0.12941 (18)	0.0375 (6)	
C8	0.7225 (2)	0.25634 (12)	0.09268 (18)	0.0369 (6)	
C9	0.6475 (2)	0.19590 (13)	0.07966 (18)	0.0377 (6)	
C10	0.7102 (3)	0.13445 (13)	0.1001 (2)	0.0439 (6)	
H10A	0.6635	0.0942	0.0915	0.053*	
C11	0.5089 (2)	0.19680 (14)	0.05067 (18)	0.0404 (6)	
C12	0.4472 (3)	0.24327 (15)	0.0931 (2)	0.0510 (7)	
H12A	0.4927	0.2780	0.1355	0.061*	
C13	0.3191 (3)	0.23809 (18)	0.0727 (3)	0.0670 (9)	
H13A	0.2792	0.2694	0.1014	0.080*	
C14	0.2502 (3)	0.18733 (19)	0.0107 (3)	0.0698 (9)	
H14A	0.1642	0.1839	−0.0018	0.084*	
C15	0.3085 (3)	0.14150 (17)	−0.0330 (2)	0.0621 (8)	
H15A	0.2619	0.1072	−0.0758	0.075*	
C16	0.4369 (3)	0.14645 (15)	−0.0133 (2)	0.0519 (7)	
H16A	0.4756	0.1154	−0.0435	0.062*	
C17	0.9245 (2)	0.32005 (12)	0.14840 (19)	0.0390 (6)	
C18	0.9904 (2)	0.34052 (13)	0.08228 (19)	0.0412 (6)	
C19	0.9824 (3)	0.30318 (15)	−0.0060 (2)	0.0488 (7)	
H19A	0.9342	0.2633	−0.0217	0.059*	
C20	1.0439 (3)	0.32469 (15)	−0.0684 (2)	0.0575 (8)	
H20A	1.0374	0.2994	−0.1263	0.069*	
C21	1.1170 (3)	0.38432 (17)	−0.0467 (3)	0.0704 (10)	
H21A	1.1590	0.3985	−0.0897	0.084*	
C22	1.1263 (3)	0.42143 (16)	0.0378 (2)	0.0639 (9)	
H22A	1.1749	0.4611	0.0517	0.077*	
C23	1.0639 (3)	0.40112 (14)	0.1047 (2)	0.0480 (7)	
C24	1.0673 (3)	0.44049 (14)	0.1899 (2)	0.0512 (7)	
H24A	1.1173	0.4797	0.2052	0.061*	
C25	1.0005 (2)	0.42356 (13)	0.2507 (2)	0.0446 (7)	
C26	0.9995 (3)	0.46893 (14)	0.3362 (2)	0.0491 (7)	
C27	1.1091 (3)	0.48489 (16)	0.4141 (2)	0.0626 (8)	
H27A	1.1851	0.4663	0.4148	0.075*	
C28	1.1067 (4)	0.52873 (18)	0.4919 (3)	0.0761 (10)	
H28A	1.1812	0.5387	0.5446	0.091*	
C29	0.9969 (5)	0.55708 (19)	0.4917 (3)	0.0830 (11)	
H29A	0.9961	0.5859	0.5444	0.100*	
C30	0.8880 (4)	0.5431 (2)	0.4138 (3)	0.0860 (12)	
H30A	0.8132	0.5638	0.4121	0.103*	
C31	0.8880 (3)	0.49803 (17)	0.3367 (3)	0.0706 (10)	
H31A	0.8128	0.4874	0.2853	0.085*	
C32	0.9292 (2)	0.36130 (13)	0.2287 (2)	0.0421 (6)	
O2A	0.6852 (4)	0.3614 (2)	−0.0778 (3)	0.0701 (8)	0.613 (3)
C33A	0.6000 (19)	0.3366 (11)	−0.0319 (16)	0.050 (3)	0.613 (3)
H33A	0.5373	0.3715	−0.0360	0.059*	0.613 (3)
H33B	0.5569	0.2965	−0.0684	0.059*	0.613 (3)
C34A	0.721 (3)	0.4298 (14)	−0.031 (2)	0.092 (3)	0.613 (3)
H34A	0.7862	0.4489	−0.0514	0.137*	0.613 (3)
H34B	0.7501	0.4254	0.0419	0.137*	0.613 (3)
H34C	0.6486	0.4596	−0.0522	0.137*	0.613 (3)
O2B	0.5987 (6)	0.4032 (3)	−0.0510 (4)	0.0701 (8)	0.387 (3)
C33B	0.625 (3)	0.3315 (18)	−0.043 (3)	0.050 (3)	0.387 (3)
H33C	0.6925	0.3201	−0.0675	0.059*	0.387 (3)
H33D	0.5506	0.3050	−0.0801	0.059*	0.387 (3)
C34B	0.732 (5)	0.438 (3)	−0.047 (4)	0.092 (3)	0.387 (3)
H34D	0.7184	0.4860	−0.0638	0.137*	0.387 (3)
H34E	0.7615	0.4158	−0.0957	0.137*	0.387 (3)
H34F	0.7927	0.4331	0.0197	0.137*	0.387 (3)
O4A	0.9249 (5)	0.2474 (3)	0.3892 (3)	0.0944 (11)	0.589 (4)
C35A	0.919 (3)	0.3217 (14)	0.387 (2)	0.062 (4)	0.589 (4)
H35A	0.8742	0.3377	0.4303	0.075*	0.589 (4)
H35B	1.0039	0.3403	0.4126	0.075*	0.589 (4)
C36A	0.8111 (9)	0.2179 (5)	0.3762 (9)	0.119 (4)	0.589 (4)
H36A	0.8222	0.1696	0.3900	0.178*	0.589 (4)
H36B	0.7558	0.2248	0.3075	0.178*	0.589 (4)
H36C	0.7751	0.2386	0.4221	0.178*	0.589 (4)
O4B	0.8506 (7)	0.2985 (3)	0.4337 (5)	0.0944 (11)	0.411 (4)
C35B	0.940 (4)	0.306 (2)	0.381 (3)	0.062 (4)	0.411 (4)
H35C	0.9674	0.2623	0.3638	0.075*	0.411 (4)
H35D	1.0128	0.3331	0.4202	0.075*	0.411 (4)
C36B	0.7513 (14)	0.2523 (8)	0.3896 (14)	0.119 (4)	0.411 (4)
H36D	0.6861	0.2599	0.4176	0.178*	0.411 (4)
H36E	0.7818	0.2061	0.4035	0.178*	0.411 (4)
H36F	0.7181	0.2595	0.3174	0.178*	0.411 (4)
O5	0.67985 (16)	0.32761 (9)	0.62699 (13)	0.0473 (4)	
O7	0.45926 (17)	0.34620 (9)	0.42540 (13)	0.0469 (4)	
C37	0.4630 (2)	0.47056 (14)	0.41811 (19)	0.0424 (6)	
C38	0.4631 (3)	0.53195 (14)	0.4654 (2)	0.0509 (7)	
H38A	0.4611	0.5724	0.4298	0.061*	
C39	0.4661 (3)	0.53555 (15)	0.5663 (2)	0.0525 (8)	
C40	0.4697 (3)	0.59979 (17)	0.6154 (3)	0.0729 (10)	
H40A	0.4653	0.6403	0.5793	0.087*	
C41	0.4795 (4)	0.6022 (2)	0.7146 (3)	0.0877 (12)	
H41A	0.4822	0.6444	0.7463	0.105*	
C42	0.4856 (4)	0.5417 (2)	0.7692 (3)	0.0863 (12)	
H42A	0.4927	0.5442	0.8372	0.104*	
C43	0.4814 (3)	0.47894 (19)	0.7251 (2)	0.0668 (9)	
H43A	0.4858	0.4392	0.7630	0.080*	
C44	0.4704 (3)	0.47424 (16)	0.6209 (2)	0.0499 (7)	
C45	0.4664 (2)	0.41001 (14)	0.57177 (19)	0.0430 (6)	
C46	0.4602 (2)	0.40943 (13)	0.47191 (19)	0.0415 (6)	
C47	0.4684 (2)	0.47056 (14)	0.31353 (19)	0.0426 (6)	
C48	0.3975 (3)	0.51715 (14)	0.2424 (2)	0.0481 (7)	
H48A	0.3425	0.5463	0.2591	0.058*	
C49	0.4071 (3)	0.52101 (16)	0.1469 (2)	0.0579 (8)	
H49A	0.3580	0.5522	0.0996	0.070*	
C50	0.4888 (3)	0.47895 (17)	0.1220 (2)	0.0623 (8)	
H50A	0.4952	0.4816	0.0577	0.075*	
C51	0.5615 (3)	0.43274 (16)	0.1919 (2)	0.0620 (8)	
H51A	0.6170	0.4042	0.1748	0.074*	
C52	0.5525 (3)	0.42866 (15)	0.2871 (2)	0.0533 (7)	
H52A	0.6028	0.3977	0.3343	0.064*	
C53	0.4726 (2)	0.34401 (14)	0.62793 (18)	0.0443 (7)	
C54	0.3679 (2)	0.32041 (16)	0.65318 (19)	0.0501 (7)	
C55	0.2527 (3)	0.3570 (2)	0.6262 (2)	0.0683 (9)	
H55A	0.2444	0.3987	0.5920	0.082*	
C56	0.1539 (3)	0.3316 (2)	0.6498 (3)	0.0872 (12)	
H56A	0.0790	0.3563	0.6321	0.105*	
C57	0.1641 (4)	0.2690 (3)	0.7004 (3)	0.0953 (13)	
H57A	0.0959	0.2522	0.7159	0.114*	
C58	0.2717 (3)	0.2326 (2)	0.7270 (3)	0.0789 (11)	
H58A	0.2768	0.1910	0.7607	0.095*	
C59	0.3778 (3)	0.25672 (17)	0.7046 (2)	0.0546 (7)	
C60	0.4909 (3)	0.21998 (16)	0.7319 (2)	0.0532 (8)	
H60A	0.4974	0.1789	0.7671	0.064*	
C61	0.5926 (3)	0.24246 (14)	0.70861 (19)	0.0442 (6)	
C62	0.5801 (2)	0.30555 (14)	0.65524 (18)	0.0416 (6)	
C63	0.7117 (3)	0.20217 (13)	0.7404 (2)	0.0449 (7)	
C64	0.7621 (3)	0.17739 (13)	0.8390 (2)	0.0498 (7)	
H64A	0.7215	0.1864	0.8851	0.060*	
C65	0.8718 (3)	0.13948 (15)	0.8696 (3)	0.0614 (8)	
H65A	0.9042	0.1230	0.9358	0.074*	
C66	0.9327 (3)	0.12617 (16)	0.8033 (3)	0.0704 (10)	
H66A	1.0071	0.1010	0.8245	0.084*	
C67	0.8848 (3)	0.14971 (17)	0.7052 (3)	0.0715 (10)	
H67A	0.9265	0.1403	0.6601	0.086*	
C68	0.7742 (3)	0.18753 (16)	0.6731 (2)	0.0596 (8)	
H68A	0.7417	0.2031	0.6064	0.072*	
O6A	0.7764 (4)	0.4266 (2)	0.7012 (3)	0.0740 (9)	0.545 (3)
C69A	0.785 (3)	0.3544 (13)	0.699 (3)	0.060 (4)	0.545 (3)
H69A	0.8590	0.3412	0.6843	0.071*	0.545 (3)
H69B	0.7922	0.3359	0.7654	0.071*	0.545 (3)
C70A	0.792 (5)	0.457 (3)	0.609 (4)	0.120 (8)	0.545 (3)
H70A	0.8107	0.5048	0.6199	0.181*	0.545 (3)
H70B	0.7152	0.4511	0.5521	0.181*	0.545 (3)
H70C	0.8594	0.4340	0.5951	0.181*	0.545 (3)
O6B	0.8608 (5)	0.3931 (3)	0.6772 (4)	0.0740 (9)	0.455 (3)
C69B	0.767 (3)	0.3710 (17)	0.711 (3)	0.060 (4)	0.455 (3)
H69C	0.7214	0.4099	0.7248	0.071*	0.455 (3)
H69D	0.8013	0.3443	0.7726	0.071*	0.455 (3)
C70B	0.816 (6)	0.441 (3)	0.601 (5)	0.120 (8)	0.455 (3)
H70D	0.8849	0.4692	0.5977	0.181*	0.455 (3)
H70E	0.7552	0.4701	0.6148	0.181*	0.455 (3)
H70F	0.7776	0.4186	0.5369	0.181*	0.455 (3)
O8A	0.2554 (2)	0.30346 (14)	0.3860 (2)	0.0771 (9)	0.883 (4)
C71A	0.3449 (8)	0.3271 (3)	0.3501 (6)	0.0604 (13)	0.883 (4)
H71A	0.3114	0.3665	0.3070	0.072*	0.883 (4)
H71B	0.3621	0.2918	0.3079	0.072*	0.883 (4)
C72A	0.2872 (8)	0.2372 (3)	0.4296 (6)	0.0946 (18)	0.883 (4)
H72A	0.2221	0.2212	0.4534	0.142*	0.883 (4)
H72B	0.3654	0.2396	0.4855	0.142*	0.883 (4)
H72C	0.2956	0.2060	0.3792	0.142*	0.883 (4)
O8B	0.3269 (18)	0.2599 (11)	0.3404 (16)	0.0771 (9)	0.117 (4)
C71B	0.332 (8)	0.345 (3)	0.337 (5)	0.0604 (13)	0.117 (4)
H71C	0.3377	0.3622	0.2734	0.072*	0.117 (4)
H71D	0.2646	0.3669	0.3523	0.072*	0.117 (4)
C72B	0.264 (8)	0.220 (3)	0.411 (5)	0.0946 (18)	0.117 (4)
H72D	0.2797	0.1716	0.4091	0.142*	0.117 (4)
H72E	0.1749	0.2281	0.3863	0.142*	0.117 (4)
H72F	0.2998	0.2363	0.4795	0.142*	0.117 (4)

### Atomic displacement parameters (Å^2^)

**Table:**

	*U*^11^	*U*^22^	*U*^33^	*U*^12^	*U*^13^	*U*^23^
O1	0.0491 (10)	0.0326 (9)	0.0388 (10)	0.0060 (8)	0.0132 (8)	0.0004 (8)
O3	0.0545 (11)	0.0461 (11)	0.0469 (11)	−0.0033 (9)	0.0190 (9)	0.0004 (9)
C1	0.0488 (17)	0.0370 (15)	0.0515 (17)	0.0037 (13)	0.0182 (14)	0.0014 (13)
C2	0.062 (2)	0.0340 (16)	0.085 (2)	0.0034 (15)	0.0225 (19)	0.0010 (16)
C3	0.069 (2)	0.0446 (19)	0.103 (3)	0.0176 (17)	0.021 (2)	0.0040 (19)
C4	0.0449 (18)	0.065 (2)	0.098 (3)	0.0163 (17)	0.0120 (18)	0.002 (2)
C5	0.0467 (18)	0.0438 (17)	0.074 (2)	0.0011 (14)	0.0138 (16)	0.0018 (16)
C6	0.0410 (15)	0.0356 (14)	0.0482 (16)	0.0025 (12)	0.0142 (13)	0.0000 (13)
C7	0.0405 (15)	0.0315 (13)	0.0408 (15)	−0.0013 (12)	0.0143 (12)	−0.0028 (12)
C8	0.0472 (16)	0.0290 (13)	0.0367 (14)	0.0048 (12)	0.0174 (12)	−0.0017 (12)
C9	0.0417 (15)	0.0368 (14)	0.0379 (14)	0.0004 (12)	0.0178 (12)	−0.0009 (12)
C10	0.0479 (17)	0.0344 (14)	0.0514 (17)	−0.0070 (13)	0.0198 (14)	−0.0047 (13)
C11	0.0433 (15)	0.0403 (14)	0.0401 (15)	−0.0014 (13)	0.0174 (13)	0.0047 (13)
C12	0.0508 (18)	0.0549 (19)	0.0480 (17)	0.0036 (15)	0.0178 (14)	−0.0034 (15)
C13	0.052 (2)	0.079 (2)	0.077 (2)	0.0111 (18)	0.0317 (18)	−0.002 (2)
C14	0.0424 (18)	0.081 (2)	0.085 (2)	−0.0039 (18)	0.0213 (18)	0.002 (2)
C15	0.0499 (19)	0.061 (2)	0.070 (2)	−0.0108 (16)	0.0135 (17)	−0.0062 (17)
C16	0.0536 (18)	0.0476 (17)	0.0575 (19)	−0.0024 (14)	0.0228 (16)	−0.0028 (15)
C17	0.0376 (14)	0.0326 (14)	0.0427 (14)	0.0004 (11)	0.0087 (12)	−0.0001 (12)
C18	0.0408 (14)	0.0336 (14)	0.0454 (15)	0.0016 (12)	0.0100 (12)	−0.0005 (12)
C19	0.0487 (16)	0.0444 (16)	0.0520 (16)	0.0000 (14)	0.0157 (14)	−0.0039 (14)
C20	0.069 (2)	0.0539 (19)	0.0529 (17)	0.0046 (17)	0.0245 (16)	−0.0044 (16)
C21	0.084 (3)	0.068 (2)	0.074 (2)	−0.0061 (19)	0.046 (2)	0.0056 (19)
C22	0.073 (2)	0.0508 (19)	0.078 (2)	−0.0146 (16)	0.0398 (19)	0.0001 (18)
C23	0.0492 (16)	0.0378 (15)	0.0565 (18)	−0.0030 (13)	0.0176 (14)	0.0005 (14)
C24	0.0504 (17)	0.0344 (15)	0.068 (2)	−0.0091 (13)	0.0196 (16)	−0.0038 (14)
C25	0.0456 (16)	0.0347 (15)	0.0486 (16)	0.0011 (12)	0.0099 (13)	−0.0006 (13)
C26	0.0592 (19)	0.0358 (15)	0.0539 (17)	−0.0024 (14)	0.0214 (15)	−0.0009 (14)
C27	0.068 (2)	0.0546 (19)	0.060 (2)	−0.0007 (16)	0.0141 (17)	−0.0113 (17)
C28	0.091 (3)	0.070 (2)	0.063 (2)	−0.011 (2)	0.020 (2)	−0.0181 (19)
C29	0.118 (3)	0.067 (2)	0.077 (2)	−0.015 (2)	0.050 (2)	−0.023 (2)
C30	0.086 (3)	0.078 (3)	0.109 (3)	0.004 (2)	0.052 (3)	−0.021 (2)
C31	0.064 (2)	0.066 (2)	0.084 (3)	−0.0064 (18)	0.0285 (19)	−0.0211 (19)
C32	0.0412 (15)	0.0368 (14)	0.0469 (16)	0.0014 (12)	0.0133 (13)	0.0027 (13)
O2A	0.095 (2)	0.061 (2)	0.0567 (18)	0.0074 (17)	0.0295 (17)	0.0117 (16)
C33A	0.062 (9)	0.043 (4)	0.045 (5)	0.014 (4)	0.021 (4)	0.000 (3)
C34A	0.099 (6)	0.073 (6)	0.099 (9)	−0.017 (5)	0.030 (5)	0.048 (5)
O2B	0.095 (2)	0.061 (2)	0.0567 (18)	0.0074 (17)	0.0295 (17)	0.0117 (16)
C33B	0.062 (9)	0.043 (4)	0.045 (5)	0.014 (4)	0.021 (4)	0.000 (3)
C34B	0.099 (6)	0.073 (6)	0.099 (9)	−0.017 (5)	0.030 (5)	0.048 (5)
O4A	0.109 (3)	0.086 (3)	0.082 (3)	−0.005 (2)	0.024 (2)	0.023 (2)
C35A	0.066 (10)	0.064 (12)	0.058 (4)	−0.004 (7)	0.023 (4)	0.009 (7)
C36A	0.108 (9)	0.109 (9)	0.136 (6)	−0.022 (5)	0.037 (6)	0.050 (7)
O4B	0.109 (3)	0.086 (3)	0.082 (3)	−0.005 (2)	0.024 (2)	0.023 (2)
C35B	0.066 (10)	0.064 (12)	0.058 (4)	−0.004 (7)	0.023 (4)	0.009 (7)
C36B	0.108 (9)	0.109 (9)	0.136 (6)	−0.022 (5)	0.037 (6)	0.050 (7)
O5	0.0445 (10)	0.0479 (11)	0.0513 (11)	−0.0031 (9)	0.0188 (9)	0.0023 (9)
O7	0.0583 (12)	0.0385 (10)	0.0408 (10)	0.0047 (9)	0.0133 (9)	−0.0031 (9)
C37	0.0453 (15)	0.0426 (15)	0.0398 (15)	0.0075 (13)	0.0155 (13)	0.0024 (14)
C38	0.064 (2)	0.0406 (16)	0.0491 (17)	0.0101 (14)	0.0212 (15)	0.0013 (14)
C39	0.0585 (19)	0.0513 (18)	0.0466 (18)	0.0085 (15)	0.0169 (15)	−0.0112 (15)
C40	0.092 (3)	0.056 (2)	0.069 (2)	0.0125 (19)	0.027 (2)	−0.0140 (18)
C41	0.114 (3)	0.074 (3)	0.069 (3)	0.017 (2)	0.025 (2)	−0.033 (2)
C42	0.112 (3)	0.098 (3)	0.048 (2)	0.033 (3)	0.027 (2)	−0.012 (2)
C43	0.079 (2)	0.078 (2)	0.0417 (18)	0.0182 (19)	0.0183 (16)	−0.0058 (18)
C44	0.0524 (17)	0.0575 (18)	0.0390 (15)	0.0119 (15)	0.0148 (14)	−0.0047 (15)
C45	0.0421 (15)	0.0488 (16)	0.0373 (15)	0.0071 (13)	0.0125 (12)	0.0011 (13)
C46	0.0434 (16)	0.0405 (15)	0.0390 (15)	0.0072 (13)	0.0119 (12)	−0.0030 (13)
C47	0.0497 (16)	0.0384 (14)	0.0418 (15)	0.0030 (13)	0.0183 (13)	0.0000 (13)
C48	0.0550 (17)	0.0435 (16)	0.0507 (17)	0.0067 (13)	0.0243 (15)	0.0079 (14)
C49	0.072 (2)	0.0544 (19)	0.0509 (18)	0.0031 (16)	0.0259 (17)	0.0111 (15)
C50	0.087 (2)	0.0595 (19)	0.0494 (18)	−0.0061 (19)	0.0357 (18)	−0.0024 (17)
C51	0.074 (2)	0.0569 (19)	0.068 (2)	0.0051 (17)	0.0414 (18)	−0.0101 (18)
C52	0.0621 (19)	0.0483 (17)	0.0528 (18)	0.0087 (15)	0.0238 (15)	−0.0004 (15)
C53	0.0481 (17)	0.0504 (17)	0.0334 (14)	0.0022 (14)	0.0127 (13)	−0.0002 (13)
C54	0.0425 (16)	0.071 (2)	0.0355 (14)	0.0030 (15)	0.0118 (12)	0.0021 (15)
C55	0.0529 (19)	0.097 (3)	0.0548 (19)	0.0117 (19)	0.0186 (16)	0.0119 (19)
C56	0.050 (2)	0.137 (4)	0.079 (2)	0.012 (2)	0.0276 (19)	0.006 (3)
C57	0.062 (2)	0.145 (4)	0.090 (3)	−0.006 (3)	0.041 (2)	0.019 (3)
C58	0.063 (2)	0.104 (3)	0.076 (2)	−0.006 (2)	0.031 (2)	0.023 (2)
C59	0.0485 (17)	0.071 (2)	0.0449 (16)	−0.0041 (16)	0.0165 (14)	0.0073 (16)
C60	0.059 (2)	0.0559 (18)	0.0451 (17)	−0.0067 (15)	0.0185 (15)	0.0058 (14)
C61	0.0492 (16)	0.0456 (16)	0.0360 (15)	−0.0040 (13)	0.0125 (13)	−0.0013 (13)
C62	0.0418 (15)	0.0489 (16)	0.0346 (14)	−0.0018 (13)	0.0141 (12)	−0.0014 (13)
C63	0.0525 (17)	0.0324 (14)	0.0494 (17)	−0.0055 (13)	0.0172 (14)	−0.0061 (13)
C64	0.0574 (18)	0.0394 (16)	0.0494 (17)	−0.0036 (14)	0.0142 (15)	−0.0040 (14)
C65	0.065 (2)	0.0397 (17)	0.066 (2)	0.0017 (16)	0.0052 (18)	−0.0009 (16)
C66	0.054 (2)	0.0440 (18)	0.105 (3)	0.0012 (15)	0.018 (2)	−0.011 (2)
C67	0.081 (3)	0.052 (2)	0.096 (3)	0.0046 (18)	0.050 (2)	−0.009 (2)
C68	0.075 (2)	0.0517 (18)	0.0574 (19)	0.0024 (17)	0.0289 (18)	−0.0020 (16)
O6A	0.070 (2)	0.070 (2)	0.079 (2)	−0.0185 (18)	0.0209 (19)	0.0046 (19)
C69A	0.048 (7)	0.065 (12)	0.060 (7)	−0.005 (6)	0.012 (4)	0.009 (8)
C70A	0.118 (14)	0.114 (18)	0.107 (8)	−0.045 (12)	0.010 (8)	0.050 (10)
O6B	0.070 (2)	0.070 (2)	0.079 (2)	−0.0185 (18)	0.0209 (19)	0.0046 (19)
C69B	0.048 (7)	0.065 (12)	0.060 (7)	−0.005 (6)	0.012 (4)	0.009 (8)
C70B	0.118 (14)	0.114 (18)	0.107 (8)	−0.045 (12)	0.010 (8)	0.050 (10)
O8A	0.0642 (17)	0.0719 (19)	0.0846 (19)	−0.0076 (14)	0.0122 (14)	0.0010 (15)
C71A	0.078 (3)	0.043 (4)	0.045 (3)	0.002 (3)	0.002 (2)	0.003 (3)
C72A	0.125 (5)	0.064 (5)	0.081 (4)	−0.041 (3)	0.018 (4)	−0.003 (3)
O8B	0.0642 (17)	0.0719 (19)	0.0846 (19)	−0.0076 (14)	0.0122 (14)	0.0010 (15)
C71B	0.078 (3)	0.043 (4)	0.045 (3)	0.002 (3)	0.002 (2)	0.003 (3)
C72B	0.125 (5)	0.064 (5)	0.081 (4)	−0.041 (3)	0.018 (4)	−0.003 (3)

### Geometric parameters (Å, °)

**Table:**

O1—C8	1.387 (3)	O5—C69A	1.37 (3)
O1—C33A	1.40 (2)	O5—C62	1.387 (3)
O1—C33B	1.51 (4)	O5—C69B	1.50 (4)
O3—C32	1.380 (3)	O7—C46	1.392 (3)
O3—C35A	1.39 (3)	O7—C71A	1.409 (8)
O3—C35B	1.50 (4)	O7—C71B	1.54 (7)
C1—C6	1.407 (4)	C37—C38	1.367 (4)
C1—C10	1.406 (4)	C37—C46	1.416 (4)
C1—C2	1.414 (4)	C37—C47	1.488 (3)
C2—C3	1.353 (4)	C38—C39	1.405 (4)
C2—H2A	0.9300	C38—H38A	0.9300
C3—C4	1.392 (5)	C39—C44	1.410 (4)
C3—H3A	0.9300	C39—C40	1.422 (4)
C4—C5	1.360 (4)	C40—C41	1.357 (5)
C4—H4A	0.9300	C40—H40A	0.9300
C5—C6	1.418 (4)	C41—C42	1.394 (5)
C5—H5A	0.9300	C41—H41A	0.9300
C6—C7	1.436 (3)	C42—C43	1.364 (5)
C7—C8	1.367 (3)	C42—H42A	0.9300
C7—C17	1.485 (3)	C43—C44	1.425 (4)
C8—C9	1.426 (3)	C43—H43A	0.9300
C9—C10	1.371 (4)	C44—C45	1.422 (4)
C9—C11	1.478 (3)	C45—C46	1.377 (3)
C10—H10A	0.9300	C45—C53	1.497 (4)
C11—C16	1.388 (4)	C47—C48	1.382 (4)
C11—C12	1.396 (4)	C47—C52	1.397 (4)
C12—C13	1.381 (4)	C48—C49	1.382 (4)
C12—H12A	0.9300	C48—H48A	0.9300
C13—C14	1.369 (4)	C49—C50	1.368 (4)
C13—H13A	0.9300	C49—H49A	0.9300
C14—C15	1.374 (4)	C50—C51	1.376 (4)
C14—H14A	0.9300	C50—H50A	0.9300
C15—C16	1.386 (4)	C51—C52	1.375 (4)
C15—H15A	0.9300	C51—H51A	0.9300
C16—H16A	0.9300	C52—H52A	0.9300
C17—C32	1.369 (3)	C53—C62	1.367 (4)
C17—C18	1.433 (3)	C53—C54	1.424 (4)
C18—C19	1.411 (4)	C54—C55	1.419 (4)
C18—C23	1.416 (4)	C54—C59	1.420 (4)
C19—C20	1.359 (4)	C55—C56	1.364 (4)
C19—H19A	0.9300	C55—H55A	0.9300
C20—C21	1.397 (4)	C56—C57	1.397 (6)
C20—H20A	0.9300	C56—H56A	0.9300
C21—C22	1.360 (4)	C57—C58	1.346 (5)
C21—H21A	0.9300	C57—H57A	0.9300
C22—C23	1.411 (4)	C58—C59	1.422 (4)
C22—H22A	0.9300	C58—H58A	0.9300
C23—C24	1.408 (4)	C59—C60	1.400 (4)
C24—C25	1.360 (4)	C60—C61	1.372 (4)
C24—H24A	0.9300	C60—H60A	0.9300
C25—C32	1.430 (4)	C61—C62	1.420 (4)
C25—C26	1.493 (4)	C61—C63	1.489 (4)
C26—C27	1.375 (4)	C63—C64	1.387 (4)
C26—C31	1.385 (4)	C63—C68	1.389 (4)
C27—C28	1.392 (4)	C64—C65	1.379 (4)
C27—H27A	0.9300	C64—H64A	0.9300
C28—C29	1.359 (5)	C65—C66	1.360 (4)
C28—H28A	0.9300	C65—H65A	0.9300
C29—C30	1.363 (5)	C66—C67	1.370 (5)
C29—H29A	0.9300	C66—H66A	0.9300
C30—C31	1.392 (5)	C67—C68	1.387 (4)
C30—H30A	0.9300	C67—H67A	0.9300
C31—H31A	0.9300	C68—H68A	0.9300
O2A—C33A	1.416 (19)	O6A—C69A	1.41 (3)
O2A—C34A	1.48 (3)	O6A—C70A	1.49 (5)
C33A—H33A	0.9700	C69A—H69A	0.9700
C33A—H33B	0.9700	C69A—H69B	0.9700
C34A—H34A	0.9600	C70A—H70A	0.9600
C34A—H34B	0.9600	C70A—H70B	0.9600
C34A—H34C	0.9600	C70A—H70C	0.9600
O2B—C33B	1.42 (3)	O6B—C69B	1.37 (4)
O2B—C34B	1.63 (4)	O6B—C70B	1.38 (7)
C33B—H33C	0.9700	C69B—H69C	0.9700
C33B—H33D	0.9700	C69B—H69D	0.9700
C34B—H34D	0.9600	C70B—H70D	0.9600
C34B—H34E	0.9600	C70B—H70E	0.9600
C34B—H34F	0.9600	C70B—H70F	0.9600
O4A—C36A	1.364 (10)	O8A—C71A	1.357 (9)
O4A—C35A	1.45 (3)	O8A—C72A	1.421 (8)
C35A—H35A	0.9700	C71A—H71A	0.9700
C35A—H35B	0.9700	C71A—H71B	0.9700
C36A—H36A	0.9600	C72A—H72A	0.9600
C36A—H36B	0.9600	C72A—H72B	0.9600
C36A—H36C	0.9600	C72A—H72C	0.9600
O4B—C36B	1.406 (17)	O8B—C72B	1.60 (7)
O4B—C35B	1.45 (4)	O8B—C71B	1.66 (5)
C35B—H35C	0.9700	C71B—H71C	0.9700
C35B—H35D	0.9700	C71B—H71D	0.9700
C36B—H36D	0.9600	C72B—H72D	0.9600
C36B—H36E	0.9600	C72B—H72E	0.9600
C36B—H36F	0.9600	C72B—H72F	0.9600
			
C8—O1—C33A	118.8 (7)	C69A—O5—C62	119.3 (13)
C8—O1—C33B	108.6 (11)	C62—O5—C69B	109.1 (13)
C32—O3—C35A	119.3 (11)	C46—O7—C71A	116.2 (3)
C32—O3—C35B	109.4 (15)	C46—O7—C71B	104 (2)
C6—C1—C10	119.1 (2)	C38—C37—C46	118.3 (2)
C6—C1—C2	119.3 (2)	C38—C37—C47	119.0 (2)
C10—C1—C2	121.6 (3)	C46—C37—C47	122.7 (2)
C3—C2—C1	120.9 (3)	C37—C38—C39	121.8 (3)
C3—C2—H2A	119.6	C37—C38—H38A	119.1
C1—C2—H2A	119.6	C39—C38—H38A	119.1
C2—C3—C4	120.1 (3)	C38—C39—C44	119.2 (2)
C2—C3—H3A	120.0	C38—C39—C40	121.2 (3)
C4—C3—H3A	120.0	C44—C39—C40	119.5 (3)
C5—C4—C3	121.0 (3)	C41—C40—C39	120.3 (3)
C5—C4—H4A	119.5	C41—C40—H40A	119.8
C3—C4—H4A	119.5	C39—C40—H40A	119.8
C4—C5—C6	120.5 (3)	C40—C41—C42	120.3 (3)
C4—C5—H5A	119.8	C40—C41—H41A	119.9
C6—C5—H5A	119.8	C42—C41—H41A	119.9
C1—C6—C5	118.2 (2)	C43—C42—C41	121.4 (3)
C1—C6—C7	119.1 (2)	C43—C42—H42A	119.3
C5—C6—C7	122.6 (2)	C41—C42—H42A	119.3
C8—C7—C6	118.9 (2)	C42—C43—C44	120.0 (3)
C8—C7—C17	120.7 (2)	C42—C43—H43A	120.0
C6—C7—C17	120.4 (2)	C44—C43—H43A	120.0
C7—C8—O1	118.1 (2)	C39—C44—C45	119.6 (2)
C7—C8—C9	123.0 (2)	C39—C44—C43	118.4 (3)
O1—C8—C9	118.9 (2)	C45—C44—C43	122.0 (3)
C10—C9—C8	116.9 (2)	C46—C45—C44	118.8 (2)
C10—C9—C11	119.5 (2)	C46—C45—C53	120.3 (2)
C8—C9—C11	123.6 (2)	C44—C45—C53	120.9 (2)
C9—C10—C1	122.9 (2)	C45—C46—O7	118.2 (2)
C9—C10—H10A	118.6	C45—C46—C37	122.1 (2)
C1—C10—H10A	118.6	O7—C46—C37	119.5 (2)
C16—C11—C12	117.7 (3)	C48—C47—C52	118.2 (2)
C16—C11—C9	120.0 (2)	C48—C47—C37	120.0 (2)
C12—C11—C9	122.0 (2)	C52—C47—C37	121.6 (2)
C13—C12—C11	120.5 (3)	C49—C48—C47	120.9 (3)
C13—C12—H12A	119.7	C49—C48—H48A	119.5
C11—C12—H12A	119.7	C47—C48—H48A	119.5
C14—C13—C12	120.8 (3)	C50—C49—C48	120.1 (3)
C14—C13—H13A	119.6	C50—C49—H49A	120.0
C12—C13—H13A	119.6	C48—C49—H49A	120.0
C13—C14—C15	119.8 (3)	C49—C50—C51	120.1 (3)
C13—C14—H14A	120.1	C49—C50—H50A	120.0
C15—C14—H14A	120.1	C51—C50—H50A	120.0
C14—C15—C16	119.9 (3)	C52—C51—C50	120.2 (3)
C14—C15—H15A	120.1	C52—C51—H51A	119.9
C16—C15—H15A	120.1	C50—C51—H51A	119.9
C15—C16—C11	121.3 (3)	C51—C52—C47	120.6 (3)
C15—C16—H16A	119.3	C51—C52—H52A	119.7
C11—C16—H16A	119.3	C47—C52—H52A	119.7
C32—C17—C18	119.1 (2)	C62—C53—C54	119.3 (3)
C32—C17—C7	120.5 (2)	C62—C53—C45	119.6 (2)
C18—C17—C7	120.3 (2)	C54—C53—C45	121.1 (2)
C19—C18—C23	118.7 (2)	C55—C54—C59	118.6 (3)
C19—C18—C17	122.2 (2)	C55—C54—C53	122.5 (3)
C23—C18—C17	119.0 (2)	C59—C54—C53	118.8 (3)
C20—C19—C18	120.9 (3)	C56—C55—C54	120.5 (3)
C20—C19—H19A	119.6	C56—C55—H55A	119.7
C18—C19—H19A	119.6	C54—C55—H55A	119.7
C19—C20—C21	120.8 (3)	C55—C56—C57	120.6 (4)
C19—C20—H20A	119.6	C55—C56—H56A	119.7
C21—C20—H20A	119.6	C57—C56—H56A	119.7
C22—C21—C20	119.6 (3)	C58—C57—C56	120.6 (3)
C22—C21—H21A	120.2	C58—C57—H57A	119.7
C20—C21—H21A	120.2	C56—C57—H57A	119.7
C21—C22—C23	121.6 (3)	C57—C58—C59	121.2 (4)
C21—C22—H22A	119.2	C57—C58—H58A	119.4
C23—C22—H22A	119.2	C59—C58—H58A	119.4
C24—C23—C22	122.5 (3)	C60—C59—C54	119.4 (3)
C24—C23—C18	119.0 (2)	C60—C59—C58	122.2 (3)
C22—C23—C18	118.4 (3)	C54—C59—C58	118.4 (3)
C25—C24—C23	122.7 (3)	C61—C60—C59	122.3 (3)
C25—C24—H24A	118.7	C61—C60—H60A	118.8
C23—C24—H24A	118.7	C59—C60—H60A	118.8
C24—C25—C32	117.7 (2)	C60—C61—C62	117.6 (3)
C24—C25—C26	121.3 (2)	C60—C61—C63	120.5 (3)
C32—C25—C26	121.0 (2)	C62—C61—C63	121.9 (2)
C27—C26—C31	118.5 (3)	C53—C62—O5	118.9 (2)
C27—C26—C25	121.1 (3)	C53—C62—C61	122.6 (2)
C31—C26—C25	120.3 (3)	O5—C62—C61	118.4 (2)
C26—C27—C28	120.3 (3)	C64—C63—C68	118.3 (3)
C26—C27—H27A	119.8	C64—C63—C61	120.0 (2)
C28—C27—H27A	119.8	C68—C63—C61	121.7 (3)
C29—C28—C27	120.8 (3)	C65—C64—C63	120.8 (3)
C29—C28—H28A	119.6	C65—C64—H64A	119.6
C27—C28—H28A	119.6	C63—C64—H64A	119.6
C28—C29—C30	119.6 (3)	C66—C65—C64	120.3 (3)
C28—C29—H29A	120.2	C66—C65—H65A	119.9
C30—C29—H29A	120.2	C64—C65—H65A	119.9
C29—C30—C31	120.3 (3)	C65—C66—C67	120.2 (3)
C29—C30—H30A	119.8	C65—C66—H66A	119.9
C31—C30—H30A	119.8	C67—C66—H66A	119.9
C26—C31—C30	120.4 (3)	C66—C67—C68	120.3 (3)
C26—C31—H31A	119.8	C66—C67—H67A	119.9
C30—C31—H31A	119.8	C68—C67—H67A	119.9
C17—C32—O3	119.2 (2)	C67—C68—C63	120.1 (3)
C17—C32—C25	122.3 (2)	C67—C68—H68A	119.9
O3—C32—C25	118.4 (2)	C63—C68—H68A	119.9
C33A—O2A—C34A	103.1 (12)	C69A—O6A—C70A	111 (2)
O1—C33A—O2A	111.0 (14)	O5—C69A—O6A	110 (2)
O1—C33A—H33A	109.4	O5—C69A—H69A	109.6
O2A—C33A—H33A	109.4	O6A—C69A—H69A	109.6
O1—C33A—H33B	109.4	O5—C69A—H69B	109.6
O2A—C33A—H33B	109.4	O6A—C69A—H69B	109.6
H33A—C33A—H33B	108.0	H69A—C69A—H69B	108.1
C33B—O2B—C34B	104 (2)	C69B—O6B—C70B	111 (3)
O2B—C33B—O1	102 (2)	O6B—C69B—O5	106 (2)
O2B—C33B—H33C	111.3	O6B—C69B—H69C	110.5
O1—C33B—H33C	111.3	O5—C69B—H69C	110.5
O2B—C33B—H33D	111.3	O6B—C69B—H69D	110.5
O1—C33B—H33D	111.3	O5—C69B—H69D	110.5
H33C—C33B—H33D	109.2	H69C—C69B—H69D	108.6
O2B—C34B—H34D	109.5	O6B—C70B—H70D	109.5
O2B—C34B—H34E	109.5	O6B—C70B—H70E	109.5
H34D—C34B—H34E	109.5	H70D—C70B—H70E	109.5
O2B—C34B—H34F	109.5	O6B—C70B—H70F	109.5
H34D—C34B—H34F	109.5	H70D—C70B—H70F	109.5
H34E—C34B—H34F	109.5	H70E—C70B—H70F	109.5
C36A—O4A—C35A	112.7 (13)	C71A—O8A—C72A	110.9 (5)
O3—C35A—O4A	109.9 (19)	O8A—C71A—O7	115.0 (5)
O3—C35A—H35A	109.7	O8A—C71A—H71A	108.5
O4A—C35A—H35A	109.7	O7—C71A—H71A	108.5
O3—C35A—H35B	109.7	O8A—C71A—H71B	108.5
O4A—C35A—H35B	109.7	O7—C71A—H71B	108.5
H35A—C35A—H35B	108.2	H71A—C71A—H71B	107.5
C36B—O4B—C35B	116 (2)	C72B—O8B—C71B	122 (4)
O4B—C35B—O3	99 (2)	O7—C71B—O8B	92 (3)
O4B—C35B—H35C	111.9	O7—C71B—H71C	113.4
O3—C35B—H35C	111.9	O8B—C71B—H71C	113.4
O4B—C35B—H35D	111.9	O7—C71B—H71D	113.4
O3—C35B—H35D	111.9	O8B—C71B—H71D	113.4
H35C—C35B—H35D	109.6	H71C—C71B—H71D	110.7
O4B—C36B—H36D	109.5	O8B—C72B—H72D	109.5
O4B—C36B—H36E	109.5	O8B—C72B—H72E	109.5
H36D—C36B—H36E	109.5	H72D—C72B—H72E	109.5
O4B—C36B—H36F	109.5	O8B—C72B—H72F	109.5
H36D—C36B—H36F	109.5	H72D—C72B—H72F	109.5
H36E—C36B—H36F	109.5	H72E—C72B—H72F	109.5

### Hydrogen-bond geometry (Å, °)

**Table:**

*D*—H···*A*	*D*—H	H···*A*	*D*···*A*	*D*—H···*A*
C3—H3A···O6A^i^	0.93	2.59	3.448 (6)	153
C43—H43A···O2B^ii^	0.93	2.57	3.304 (7)	136
C51—H51A···O1	0.93	2.38	3.233 (3)	152

Symmetry codes: (i) −*x*+2, *y*−1/2, −*z*+1; (ii) *x*, *y*, *z*+1.
